# Silk Fibroin Nanofibers: Advancements in Bioactive Dressings through Electrospinning Technology for Diabetic Wound Healing

**DOI:** 10.3390/ph17101305

**Published:** 2024-09-30

**Authors:** Afaf Aldahish, Nirenjen Shanmugasundaram, Rajalakshimi Vasudevan, Taha Alqahtani, Saud Alqahtani, Ahmad Mohammad Asiri, Praveen Devanandan, Tamilanban Thamaraikani, Chitra Vellapandian, Narayanan Jayasankar

**Affiliations:** 1 Department of Pharmacology, College of Pharmacy, King Khalid University, Abha, Saudi Arabia; 2 Department of Pharmacology, SRM College of Pharmacy, SRM Institute of Science and Technology, Kattankulathur, Chengalpattu 603203, India; 3 Khamis Mushayt General Hospital, Aseer Health Cluster, Ministry of Health, Khamis Mushait 62433, Saudi Arabia; 4 Department of Pharmacy Practice, St. Peter’s Institute of Pharmaceutical Sciences, Vidya Nagar, Hanamkonda 506001, India

**Keywords:** diabetic wounds, wound healing, bioactive dressings, polymeric nanofibers, silk biomaterials, electrospinning, silk fibroin, drug delivery

## Abstract

Background: Non-healing diabetic wounds represent a significant clinical challenge globally, necessitating innovative approaches in drug delivery to enhance wound healing. Understanding the pathogenesis of these wounds is crucial for developing effective treatments. Bioactive dressings and polymeric nanofibers have emerged as promising modalities, with silk biomaterials gaining attention for their unique properties in diabetic wound healing. Purpose of Review: The purpose of this review is to examine the challenges and innovations in treating non-healing diabetic wounds, emphasizing the global burden and the need for effective solutions. This review explores the complex mechanisms of wound healing in diabetes and evaluates the therapeutic potential of bioactive dressings and polymeric nanofibers. Special focus is given to the application of silk biomaterials, particularly silk fibroin, for wound healing, detailing their properties, mechanisms, and clinical translation. This review also describes various nanofiber fabrication methods, especially electrospinning technology, and presents existing evidence on the effectiveness of electrospun silk fibroin formulations. Recent Findings: Recent advancements highlight the potential of silk biomaterials in diabetic wound healing, owing to their biocompatibility, mechanical strength, and controlled drug release properties. Electrospun silk fibroin-based formulations have shown promising results in preclinical and clinical studies, demonstrating accelerated wound closure and tissue regeneration. Summary: Non-healing diabetic wounds present a significant healthcare burden globally, necessitating innovative therapeutic strategies. Bioactive dressings and polymeric nanofibers, particularly silk-based formulations fabricated through electrospinning, offer promising avenues for enhancing diabetic wound healing. Further research is warranted to optimize formulation parameters and validate efficacy in larger clinical trials.

## 1. Introduction

Diabetes mellitus (DM) is a widespread and impactful metabolic disorder marked by chronic hyperglycemia due to defects in insulin secretion or action [1]. This persistent elevation of blood glucose levels leads to a spectrum of complications, one of the most problematic being non-healing diabetic wounds [2]. These chronic wounds, particularly those occurring in the lower extremities, pose substantial challenges in patient care due to their tendency to develop infections, delay in healing, and risk of progression to severe outcomes such as amputation. Understanding the pathophysiological mechanisms behind these non-healing wounds is crucial for developing effective therapeutic strategies [3]. The healing of diabetic wounds is hindered by a range of factors related to the underlying pathophysiology of diabetes. Persistent inflammation is another critical factor that obstructs the healing process. Chronic inflammation in diabetic wounds is driven by sustained high levels of pro-inflammatory cytokines and oxidative stress. This constant inflammatory state can lead to further tissue damage and delays in the resolution of the inflammatory phase, thereby prolonging the healing process [4].

The global burden of non-healing diabetic wounds is substantial and growing. As of 2019, approximately 463 million people worldwide were diagnosed with diabetes. With the global prevalence of diabetes expected to rise to 700 million by 2040, the incidence of diabetic wounds is also projected to increase significantly [5]. This rise in diabetic cases is accompanied by an increased prevalence of chronic wounds, which impose a considerable economic burden on healthcare systems and adversely affect patients’ quality of life. The economic impact of diabetic wounds extends beyond direct medical costs, such as expenses for hospitalizations, treatments, and medications. It also includes indirect costs related to loss of productivity, long-term care needs, and diminished quality of life for patients [6]. Non-healing wounds often lead to frequent hospital visits, prolonged treatment durations, and higher rates of morbidity and mortality. Consequently, addressing this health crisis requires innovative and effective solutions to improve wound management and patient outcomes [7].

Traditional wound care methods often fall short when dealing with the complexities of diabetic wound healing. Common dressings such as gauze, semi-permeable films, foam, and hydrogel dressings have various limitations. For instance, gauze dressings may not adequately absorb wound exudate or maintain a moist environment, which is critical for promoting healing. Semi-permeable films and foam dressings can sometimes cause discomfort or lead to infection if not properly managed. Hydrogel dressings, while beneficial in maintaining moisture, may not always provide the sustained release of therapeutic agents needed for effective wound management [8]. To address these shortcomings, there is a growing need for advanced wound dressings that offer controlled and sustained release of therapeutic agents while creating an optimal environment for healing. Recent advancements in wound care technology have focused on developing bioactive dressings and polymeric nanofibers, which have shown promising results in enhancing drug delivery and supporting wound healing processes. Among the innovative approaches, Electrospun nanofibers, particularly those derived from silk fibroin, have emerged as a highly promising solution for diabetic wound care. Electrospinning technology allows for the precise fabrication of nanofibers with customizable properties, such as fiber diameter, porosity, and surface chemistry. These nanofibers exhibit a high surface area-to-volume ratio, which enhances their ability to deliver therapeutic agents effectively [9].

Silk fibroin, in particular, is known for its biocompatibility, biodegradability, and excellent mechanical properties. When used in electrospun nanofibers, silk fibroin provides a supportive scaffold that mimics the extracellular matrix, facilitating cell attachment, proliferation, and migration. This scaffold structure also allows for the continuous release of drugs and bioactive factors, thereby addressing the limitations of traditional dressings. The unique properties of electrospun nanofibers enable them to create a conducive microenvironment for wound healing. They support essential processes such as tissue regeneration, angiogenesis, and exudate absorption [10]. Furthermore, the ability to load nanofibers with various therapeutic agents, including growth factors, anti-inflammatory agents, and antimicrobial agents, enhances their effectiveness in managing diabetic wounds. The challenge of non-healing diabetic wounds highlights the need for advanced therapeutic strategies and innovative wound care technologies [11]. The limitations of traditional wound care methods necessitate the development of more effective solutions. Electrospun nanofibers, especially those made from silk fibroin, offer a promising advancement in wound care technology. Their ability to provide controlled drug release, support tissue regeneration, and enhance the healing environment positions them as a valuable tool in addressing the global burden of diabetic wounds.

The purpose of this Comprehensive review is to examine the challenges and innovations in treating non-healing diabetic wounds, emphasizing the global burden and the need for effective solutions. This review explores the complex mechanisms of wound healing in diabetes and evaluates the therapeutic potential of bioactive dressings and polymeric nanofibers. Special focus is given to the application of silk biomaterials, particularly silk fibroin, for wound healing, detailing their properties, mechanisms, and clinical translation. This review also describes fabrication methods, especially electrospinning technology, and presents existing evidence on the effectiveness of electrospun silk fibroin formulations.

## 2. Pathogenesis and Intricate Mechanisms of Non-Healing Diabetic Wounds

Wound healing is a complex, highly intricate biological process, activated in response to compromise of the integrity of tissue, which leads the series of cellular and molecular events through a well-coordinated sequence aimed at restitution of the structure and function of damaged tissue back to normal. This is typically divided into four different stages: Hemostasis, inflammation, proliferation, and remodeling. First, there is the stage of hemostasis mediated by platelets. These, upon contact with the exposed collagen of the injured tissue, are activated, aggregate, and adhere to the distorted endothelium, thereby triggering the coagulation cascade. This leads to the conversion of fibrinogen to fibrin and, hence, the formation of a thrombus that provides a provisional extracellular matrix. At the same time, activated platelets express proteins that promote migration and adhesion of neutrophils and monocytes to the site of injury. In addition, these platelets produce growth factors, including TGF-β (transforming growth factor -β) and PDGF (platelet-derived growth factor), required in the healing process [12] and the role of the various growth factors in wound healing is given in Table 1.

Following hemostasis, there is the inflammatory phase characterized by infiltration of cells of inflammation to the wound site. Neutrophils, as the first responders, assume the role for tissue debridement and control of infection. The process entered into by these cells is: cells adhere to the endothelium near the area of wounding and then cross damaged capillaries or move across intercellular gaps of endothelial cells in a process called diapedesis. They also secrete growth factors, which stimulate cell growth and proteases, which digest extracellular matrix components, thereby further promoting wound healing. As the inflammatory process chronicles, circulating monocytes infiltrate into the tissue and mature into macrophages. These macrophages are polarized first into a pro-inflammatory M1 phenotype and participate in the phagocytosis of the wound, clearing it of pathogens, foreign bodies, apoptotic neutrophils, and damaged tissue. They also aid in the production of cytokines and other mediators that sustain the inflammatory response. Soon after tissue injury, the resident mast cells respond by liberating proteases that hydrolyze the matrix and cytokines involved in recruiting neutrophils. Later on, this is coordinated by T-lymphocytes that come later to coordinate tissue remodeling. As inflammation declines, the healing process enters into its proliferative phase, characterized by a change in macrophage phenotype from M1 to M2. In essence, the underlying process is determined by the interplay of complex molecular and cellular mechanisms where the macrophage changes from a pro-inflammatory to anti-inflammatory role. During this phase, M2 macrophages release growth factors such as VEGF and TGF-β, which are involved in the cell proliferation and protein synthesis. They also secrete protease inhibitors, proteases, and anti-inflammatory mediators that promote the formation of granulation tissue. Further subtypes of the M2 macrophages include M2a, M2b, and M2c subtypes, all of which have their role distinct in this proliferative phase. M2a macrophages, polarized by IL-4 and IL-13, produce fibroblasts and extracellular matrix components. In contrast, M2b macrophages produce IL-10, TNF, and IL-6, which have an anti-inflammatory effect. It is polarized by M2c macrophages that secrete vascular structure and extracellular matrix remodeling in this phase, thanks to IL-10 and TGF-β. This process is accompanied by a characteristic process called angiogenesis or the formation of new blood vessels, which is driven by proangiogenic factors of which FGF-2 and VEGF are examples. This newly formed capillary network provides oxygen and nutrition for the rapidly growing cells of healing wound. Meanwhile, proliferating capillaries account for the granular nature of granulation tissue in appearance, which acts as a scaffolding for keratinocyte migration to re-epithelialize the wound [13,14].

**Figure 1:**
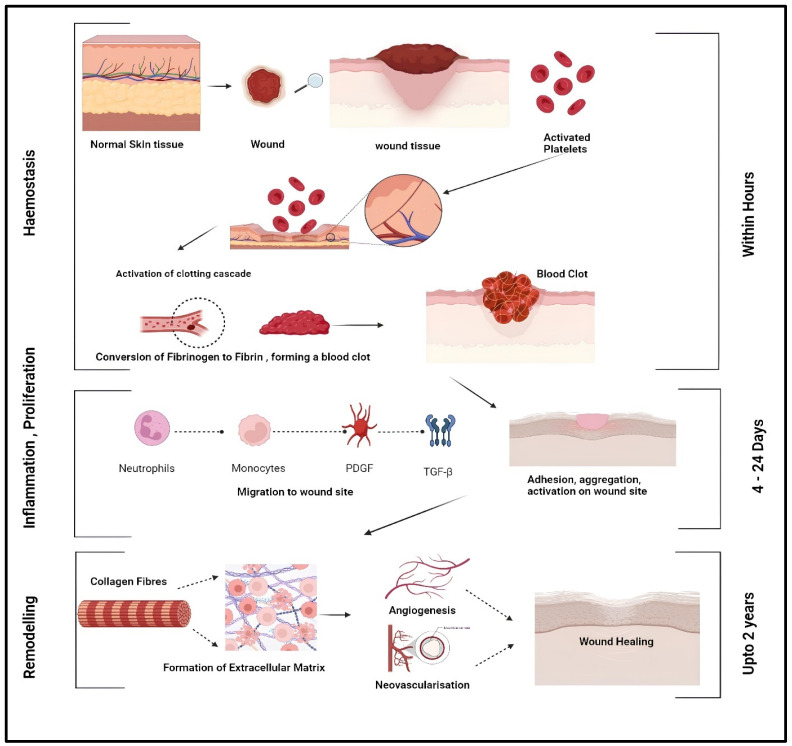
General Mechanisms of Wound Healing [26]—Wound healing progresses through three phases. The inflammatory phase starts with clot formation and immune response to prevent infection. During the proliferative phase, new tissue and blood vessels develop to restore the wound. The remodeling phase then involves the strengthening and reorganization of the new tissue for final repair and recovery.

**Figure 2:**
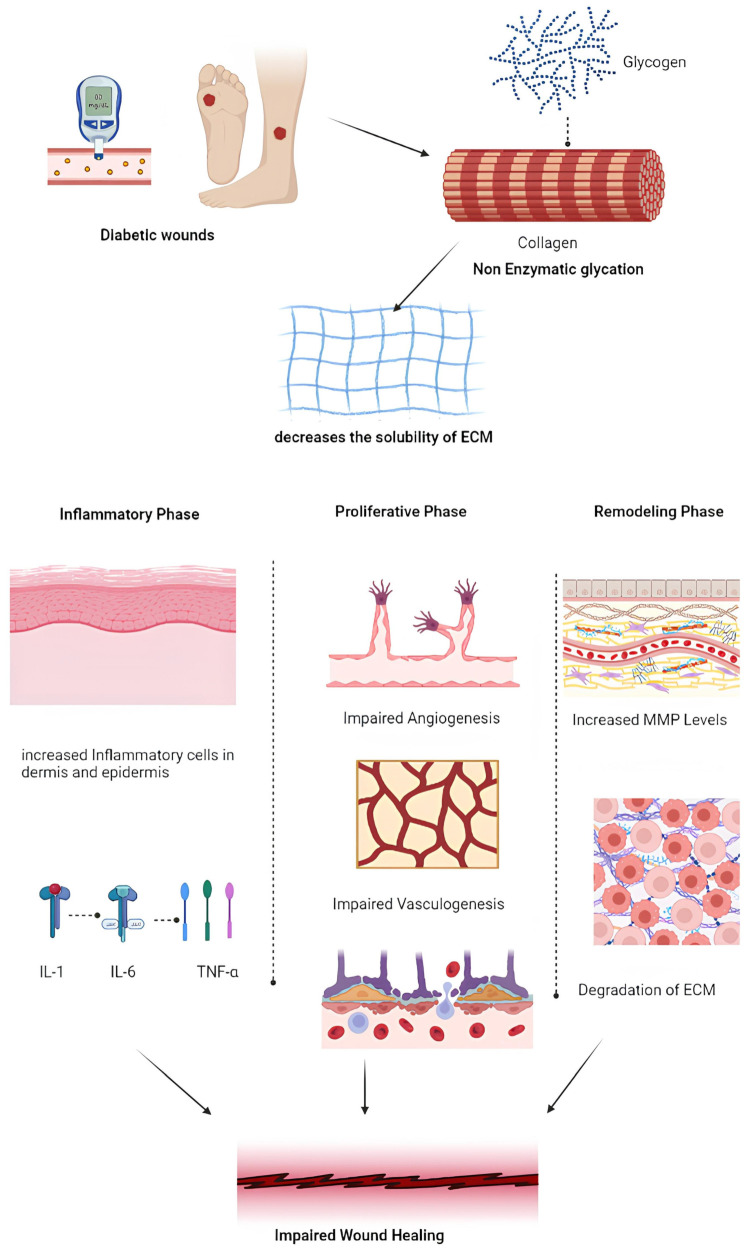
In diabetic wounds, impaired wound healing is primarily due to decreased collagen synthesis, which reduces the solubility of the extracellular matrix (ECM) and triggers an enhanced inflammatory response [26]. During the inflammatory phase, pro-inflammatory cytokines such as IL-1, IL-6, and TNF-α are released. The proliferative phase is characterized by impaired angiogenesis and vasculogenesis, while in the remodeling phase, increased levels of matrix metalloproteinases (MMPs) lead to further degradation of the ECM, ultimately contributing to impaired wound healing. (IL—interleukin, TNF—tumor necrosis factor, MMP—matrix metalloproteinase, and ECM—extracellular matrix).

The final stage is the remodeling phase, after the initial injury in between two to three weeks, when granulation tissue is gradually replaced by scar tissue. The blood vessels become less dense in number and collagen fibers are arranged and altered. There is continuous synthesis and breakdown of collagen and that is in turn controlled by matrix metalloproteinases. Through cross-linking and alignment of collagen fibers along lines of tension, tensile strength of the wound is achieved. Wound contraction also takes place where myofibroblasts draw the edges of the wound closer thereby decreasing the size of the wound [15,16]. Ultimately, it is the highly coordinated and dynamic process involving multiple cellular and molecular mechanisms which goes into the restoration of tissue integrity and function as clearly depicted in Figure 1. While the general process of wound healing is a well-coordinated sequence of events, the presence of certain pathological conditions can significantly alter this process. One such condition is diabetes, which profoundly impacts wound healing dynamics. Diabetic wounds, particularly in the context of chronic hyperglycemia, present a unique set of challenges that disrupt the normal wound healing cascade. Understanding the specific pathogenesis of diabetic wound healing is crucial, as it sheds light on the mechanisms that lead to delayed wound closure and chronic wound formation in diabetic patients as shown in Figure 2. Diabetic wounds face numerous challenges that significantly impede the healing process. A key issue is vasculopathy, where macrovascular complications in diabetes hinder the delivery of oxygen and essential nutrients to the wound site [17,18]. This is compounded by microcirculatory deficiencies, such as basement membrane thickening and reduced capillary size, which restrict physiological exchanges and impair blood flow. The situation is further exacerbated by endothelial dysfunction, which limits the production of nitric oxide, leading to suboptimal blood flow and delayed healing [19]. Another critical factor is neuropathy, where disruptions in motor, autonomic, and sensory nerve fibers diminish the ability to sense external stimuli, such as heat and pressure, delaying the healing process [20]. This sensory loss, coupled with motor fiber defects, increases physical stress on the wound, making it more prone to bacterial infections. Infections are a major concern in diabetic wounds, often leading to severe complications like abscesses, osteomyelitis, and cellulitis. These infections can rapidly develop in the hypoxic environment of a diabetic wound, further delaying the healing process [21,22,23]. The immune system also plays a role in delayed healing, as diabetes weakens immune cells, reducing their ability to combat infections. High blood sugar levels further inhibit immune function, increasing the risk of complications like sepsis or gangrene [24]. Additionally, interrupted growth factor activity is a significant contributor to delayed healing in diabetic wounds. Growth factors such as FGF, TGF-β and VEGF are critical for wound healing, but their levels are markedly reduced in diabetic patients, leading to prolonged inflammation and delayed tissue development [25]. Cellular dysfunction in key skin cells, including keratinocytes and fibroblasts, further hinders the healing process. The presence of specific microRNAs in diabetic foot ulcer fibroblasts has been shown to impair cellular functions, making wound recovery even more challenging [26]. Understanding the pathology of wound healing and the specific challenges presented by diabetic wound healing provides a foundational context for exploring the critical role of wound dressings. These dressings are not merely supplementary but play a pivotal role in managing wound healing by addressing the unique needs and complexities associated with different types of wounds, particularly those complicated by diabetes.

## 3. Role of Bioactive Wound Dressings and Its Types

**Figure 3:**
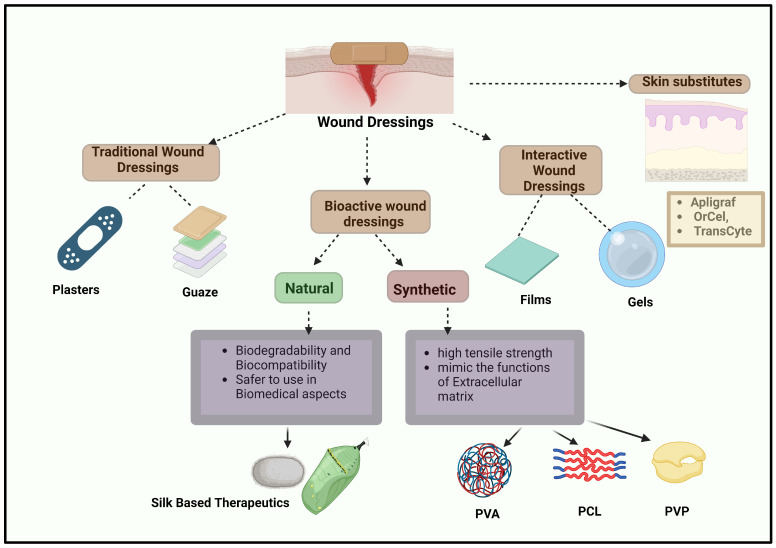
Overview of Wound Dressing Types and Their Characteristics: This figure categorizes wound dressings into traditional, interactive, and bioactive types. Traditional dressings, like plasters and gauze, offer basic protection. Interactive dressings (films, gels) and bioactive dressings (natural and synthetic) enhance healing. Natural materials, such as silk-based therapeutics, provide biodegradability and biocompatibility, while synthetic materials like PVA, PCL, and PVP offer high tensile strength. Skin substitutes like Apligraf, OrCel, and TransCyte represent advanced wound care options.

Wound dressings are integral to the effective management of various injuries, primarily by shielding wounds from bacterial invasion and expediting the process of wound healing [27]. Despite the availability of numerous wound dressings, some currently used materials exhibit limitations such as delayed healing, inadequate gaseous permeability, insufficient moisture retention, and the potential to trigger allergic reactions [28]. These shortcomings underscore the urgent need for the development of more effective wound dressing materials. Wound dressings are broadly categorized into four types: traditional/passive dressings, interactive materials, skin substitutes, and bioactive dressings as shown in Figure 3 [29]. Traditional dressings, such as wool dressings, plasters, gauze, and bandages, primarily function to protect the injury from foreign contaminants, control bleeding, cushion the wound, and absorb exudate. However, these dressings have limitations, including the risk of wound exudate leakage, which can lead to bacterial infections, and the potential for skin damage during removal.

Interactive dressings, which include composites, films, gels, foams, and sprays, offer a more advanced approach to wound care by providing a moist environment conducive to healing enhancing re-epithelialization and granulation and promoting water transmission. These dressings can also be infused with bioactive agents to further accelerate the healing process. Skin substitutes, such as OrCel, Apligraf, and TransCyte, are sophisticated tissue-engineered structures typically developed from cell co-cultures or cell-seeded scaffolds. These substitutes are particularly effective in skin regeneration. However, they come with challenges, such as the risk of wound infections, disease transmission, potential rejection by the body, high costs, and limited shelf life [30]. Dermal grafts, including acellular xenografts, autografts, and allografts, are essential in dermatology and plastic surgery for treating traumatic wounds, burn reconstruction, post-oncologic resection defects, vitiligo, scar contracture release, hair restoration, and congenital skin deficiencies. Despite their utility, these materials are not suitable for managing complex injuries, particularly those involving exposed bones or deep tissue spaces [31]. Bioactive dressings, such as hydrocolloids, sponges, wafers, foams, nanofibers, hydrogels, and collagen-based materials, represent a significant advancement in wound care. These dressings are biodegradable, biocompatible, and can serve as drug delivery systems for therapeutic agents, including nanoparticles, growth factors, vitamins, and antibiotics, all of which contribute to improved wound healing [32]. Nanofibers [33] and hydrogels [34], in particular, stand out for their ability to be loaded with drugs and provide controlled delivery of bioactive agents, thus categorizing them as bioactive wound dressings.

While these wound dressings have played a significant role in wound management, the advent of advanced materials has led to the development of more sophisticated options. Among these, polymeric nanofibers have emerged as a promising class of wound dressings, offering unique properties that enhance healing outcomes. These nanofibers not only provide a physical barrier but also deliver therapeutic agents in a controlled manner, making them particularly suitable for addressing the complex needs of modern wound care [35]. Building on the foundation of bioactive wound dressings, the incorporation of polymeric nanofibers offers a promising approach to further enhance the therapeutic potential of these dressings, particularly by enabling controlled drug delivery, improved mechanical properties, and better integration with the wound microenvironment.

## 4. Mechanical Properties and Biocompatibility in Wound Dressings

As wound dressing technologies continue to evolve, the integration of advanced materials, particularly polymeric nanofibers, has opened new avenues for enhancing wound care. However, to fully realize the therapeutic potential of these innovations, it is essential to consider not only their ability to deliver bioactive agents but also their inherent mechanical properties and biocompatibility. The mechanical properties of wound dressing materials play a pivotal role in their effectiveness in promoting wound healing. Tensile strength, elasticity, porosity, permeability, flexibility, and adhesion are key factors that determine the material’s performance. Tensile strength ensures the material can withstand mechanical stress without breaking, contributing to the durability of the dressing during wound care. Elasticity is critical for the material to conform to the wound bed and accommodate movement, especially in dynamic areas. Adequate porosity and permeability allow for oxygen exchange and moisture management while preventing bacterial invasion, maintaining an optimal healing environment. Flexibility is essential to ensure patient comfort and to avoid causing additional trauma to the wound during movement. Adhesion properties should enable the dressing to adhere securely to the wound bed without causing excessive pain or damage during dressing changes. In addition to mechanical properties, several parameters influence the wound healing process. Biocompatibility is a crucial consideration, as the material must not elicit an immune response or cause cytotoxicity. The degradation rate of biodegradable materials should be in harmony with tissue regeneration, as rapid degradation can result in premature loss of material, while slow degradation may cause irritation. Moisture retention capabilities are vital for maintaining the appropriate hydration levels in the wound, fostering cellular migration and proliferation. Materials designed for drug delivery can incorporate therapeutic agents such as antimicrobial peptides or growth factors, which directly address infection and promote tissue regeneration. Furthermore, material-cell interactions, influenced by surface properties such as roughness and charge, can enhance cell attachment, migration, and proliferation, contributing to effective wound closure. Some advanced materials may even provide mechanical stimulation to the wound, which can enhance cellular activity and collagen deposition, further accelerating the healing process. Considering both mechanical properties and these influential parameters allows for the design of wound dressings that not only protect but actively promote healing [36].

## 5. Polymeric Nanofibers Used in Wound Dressings

Polymeric nanofibers have emerged as a promising tool in the treatment of diabetic wounds due to their unique properties and potential to enhance wound healing. Among the various materials explored, silk fibroin stands out due to its exceptional biocompatibility and mechanical strength. Understanding the broader landscape of polymeric nanofibers, including both synthetic and natural variants, is crucial to appreciating the specific advantages silk fibroin offers in wound healing applications. These nanofibers, typically fabricated using both synthetic polymers such as polylactic acid (PLA), polycaprolactone (PCL), and polyvinyl alcohol (PVA), as well as natural polymers like chitosan, collagen, and gelatin, are characterized by their high surface area-to-volume ratio, porosity, and ability to mimic the extracellular matrix (ECM) [37]. Natural polymers offer additional benefits such as biocompatibility and bioactivity, which are particularly advantageous in promoting cell adhesion, proliferation and Natural polymers typically exhibit minimal immune response, while synthetic polymers are often preferred for their ease of electrospinning and excellent mechanical properties, including flexibility and stiffness The combination of these materials in nanofiber form supports cellular attachment, proliferation, and migration- key processes in wound healing [38,39]. Moreover, polymeric nanofibers can be engineered to incorporate and gradually release therapeutic agents directly at the wound site, ensuring sustained delivery of active ingredients, improving the healing process, and reducing infection risks [40]. The structure of these nanofibers closely resembles the ECM, providing a scaffold for tissue regeneration and enhancing natural healing processes, which are often impaired in diabetic patients. Additionally, polymeric nanofibers can promote angiogenesis by delivering factors such as VEGF, improve moisture retention, and can be functionalized with antimicrobial agents to prevent bacterial colonization. Despite the promising potential, challenges remain, including issues of biocompatibility, biodegradability, and large-scale production, which future research will need to address to realize the full potential of nanofiber-based therapies in clinical settings [39,41,42]. The selection of polymers for nanofiber-based wound dressings is not governed by a universal standard but is rather dictated by the specific functions desired from the nanofibers. The choice of polymers significantly influences properties such as the viscosity of the spinning solution, morphology of the nanofibers, mechanical strength, biocompatibility, and physicochemical characteristics [43]. Polymers used in this context are generally categorized into synthetic and natural types, each offering distinct advantages. To delve into the synthetic polymers utilized in wound dressings, The following are the synthetic polymers used in Bioactive wound dressings intended for Diabetic wound healing and Table 2 summarizes the various synthetic polymer formulations and their experimental applications in wound healing studies. By highlighting the strengths of synthetic polymers, we can better understand the context in which silk fibroin can be integrated as a viable alternative or complement to these materials.

Poly(lactic-co-glycolic acid) (PLGA) is a biocompatible and biodegradable polymer synthesized from lactic acid and glycolic acid. It is widely recognized for its excellent mechanical strength and flexibility, making it suitable for nanofibrous wound dressing applications. The degradation rate of PLGA can be adjusted by modifying the ratio of lactic acid to glycolic acid, molecular weight, and crystallinity [56]. However, PLGA’s poor water solubility often necessitates the use of organic solvents, which can introduce cytotoxicity and hinder diabetic wound healing. To address this, modifications such as the synthesis of N-carboxyethyl PLGA have been explored to enhance water solubility. Additionally, the inclusion of chitosan and recombinant human platelet-derived growth factor in PLGA-based nanofibers has been shown to increase hydrophilicity, promoting wound healing in diabetic models [57]. The study by Castro et al. focuses on creating electrospun fibrous membranes made from β-tricalcium phosphate (β-TCP) and Poly(lactic-co-glycolic acid) (PLGA) for guided bone regeneration. The research demonstrates that combining the biodegradable nature of PLGA with the osteoconductive properties of β-TCP results in membranes that effectively support bone tissue regeneration [58]. Poly(lactic acid) (PLA) is a thermoplastic aliphatic polyester derived from renewable resources. Widely used in pharmaceuticals and medical devices, PLA offers biocompatibility and mechanical strength [59,60]. However, PLA may exhibit poor compatibility with blood, a limitation that can be mitigated by blending it with other polymers like chitosan derivatives. This blending enhances its blood compatibility, making PLA a valuable component in wound healing platforms that support cell attachment and proliferation. The work carried out by Chengyi Liu and colleagues details the development of a polylactic acid (PLA) electrospun wound dressing, modified with polyethylene glycol (PEG), rosmarinic acid, and graphite oxide. These modifications were aimed at enhancing the dressing’s properties—PLA improved its flexibility and biocompatibility, rosmarinic acid contributed antioxidant properties, and graphite oxide increased mechanical strength. Through careful fabrication and characterization, the study demonstrates that this multifunctional wound dressing shows significant potential for promoting wound healing, making it a promising option for advanced wound care [61]. Poly(ε-caprolactone) (PCL) is another biocompatible and biodegradable polymer known for its functional ester bonds, which allow degradation into non-toxic byproducts in physiological conditions [62]. Despite its ability to mimic the extracellular matrix (ECM), PCL’s hydrophobicity can limit cell attachment and proliferation [63]. To overcome this, PCL is often combined with hydrophilic polymers such as collagen, gelatin and chitosan. The research conducted by Deiviga Murugan et al. presents an innovative approach to wound healing with poly(ε-caprolactone) (PCL) nanofibrous scaffolds infused with the homeopathic mother tincture of *Syzygium cumini*. The study emphasizes PCL’s pivotal role in providing structural stability and mechanical strength to the scaffolds. Its biocompatibility and controlled degradation characteristics complement the healing properties of *Syzygium cumini*, creating a synergistic effect that enhances wound healing outcomes [64]. Polyvinyl alcohol (PVA) is a water-soluble, non-toxic polymer with excellent mechanical properties and chemical resistance [65]. Its flexibility and swelling capacity make it a strong candidate for wound dressings. However, PVA tends to be unstable in aqueous environments, leading researchers to explore crosslinking, grafting and copolymerization with other polymers like polyvinyl pyrrolidone (PVP) and sterculia to enhance stability [66]. PVA-based nanofibers have also been incorporated with silver nanoparticles to provide antibacterial properties, which are critical for wound healing [67]. The study by Fatahian et al. explores the fabrication of electrospun polyvinyl alcohol (PVA) nanofibers with antibacterial and hemostatic properties for wound healing applications. The researchers developed a novel PVA-based nanofiber scaffold designed to offer both antimicrobial protection and promote blood clotting. This dual-functionality aims to enhance wound management by providing a protective barrier against infections while facilitating rapid Hemostasis [68].

Natural polymers are biopolymers derived from living organisms and have been widely used in various biomedical applications due to their inherent biocompatibility and biodegradability [69]. These polymers, which include chitosan, alginate, collagen, and silk fibroin, offer unique advantages in tissue engineering and wound care [70]. They are often favored for their ability to interact favorably with biological tissues, support cell growth, and promote healing processes. However, each natural polymer has its limitations, such as varying mechanical properties, degradation rates, and ease of processing. Among natural polymers, silk is distinguished by its exceptional biocompatibility, biodegradability, and mechanical strength. Its unique porous structure enhances cellular infiltration and tissue regeneration, addressing many of the limitations associated with other natural polymers and making it a valuable material for advanced wound care applications [71].

## 6. Silk Biomaterials and Silk Based Therapeutics in Diabetic Wound Healing

Silks are polymer derived proteins produced by various insects, including silkworms, spiders, bees, and others. Among these, silkworm silk, particularly from the domesticated species *Bombyx mori* (*B. mori*), is the most extensively studied and commercially utilized due to its favorable properties and availability [72]. Silks can be broadly categorized into mulberry and non-mulberry types, with mulberry silk being derived from the domesticated *Bombyx mori* silkworms. Non-mulberry silks, also known as wild silks, are produced by various species within the Saturniidae family, such as *Antheraea mylitta* (Tasar), *Antheraea assama* (Muga), and *Samia ricini* (Eri) [73,74]. These wild varieties differ in fibroin sequences and structural properties, leading to variations in mechanical strength, bioactivity, and degradation behavior. Spider silk, produced by species such as *Nephila clavipes* and *Araneus diadematus*, is renowned for its exceptional mechanical strength, surpassing that of silkworm silk. However, the production of spider silk on a commercial scale is challenging due to its heterogeneous nature and the difficulty in obtaining sufficient quantities [75,76,77]. As a result, *Bombyx mori silk* remains the primary source for silk-based biomaterials.

### 6.1. Silk Fibroin’s Structure and Properties

Silk from the *Bombyx mori* silkworm comprises two primary proteins: fibroin and sericin. Fibroin, a core protein surrounded by sericin proteins [78]. The repetitive hydrophobic sequences within fibroin, mainly composed of glycine and alanine, contribute to its high strength and durability, making it a desirable biomaterial [79]. Fibroin, which constitutes approximately 70% of the silk’s weight, forms the inner core of the silk thread, providing mechanical strength. The remaining 30% is composed of sericin, a protein that surrounds the fibroin core in a thin layer and also contains fat/wax (0.8–1%) and color/ash (1–1.4%) [80]. Silk, a natural protein polymer, is recognized by the U.S. Food and Drug Administration (FDA) for medical applications due to its biocompatibility and unique properties [81]. Silk fibroin is derived from mulberry silk after the removal of the outer sericin layer, which is known to trigger an immune response when combined with fibroin. During the spinning process, the silkworm larvae extrude two fine fibroin filaments (approximately 10 µm in diameter) through their spinnerets, which are simultaneously coated with sericin from the silk glands. Upon exposure to air, these protein fibers become stronger and tougher [82,83].

The major structural component of silk, fibroin, contributes to its mechanical strength, while sericin acts as a glue-like coating that holds the fibroin filaments together. Each silk Fiber consists of two fibroin filaments encased in sericin [84]. These nanofibrils are the fundamental building blocks of silk and are tightly intertwined to form larger fibril units known as microfibrils, which range from 20 to 200 nm in diameter. Both microfibrils and nanofibrils are aligned parallel to the silk fibroin filaments, contributing to the silk’s robust fibrous properties [85,86]. Silk fibroin’s molecular structure consists of polypeptide chains with molecular weights ranging from 200 to 350 kDa. The primary structure of fibroin is an H-L complex, which includes a heavy (H) chain polypeptide and a light (L) chain polypeptide connected by a single disulfide bond at the C-terminus of the H-chain. Additionally, a glycoprotein called P25 is noncovalently bound to the H-L chains, playing a crucial role in maintaining the overall structural integrity of silk fibroin. The H-chain is largely responsible for the fibrous properties of silk, with glycine (46%), serine (12%), and alanine (30%) being the most prevalent amino acids in this chain. In contrast, the non-fibrous L-chains contain amino acids such as isoleucine, leucine, valine, and other acidic amino acids [87]. Owing to its biocompatibility, gradual degradation, low immunogenicity, versatility, and excellent mechanical properties, silk fibroin is increasingly being explored for various biomedical applications. Its ability to adapt to biological environments suggests that it may integrate better into the body with potentially reduced material-associated thrombosis [88,89,90]. This potential pave the way for developing biomaterials with tailored properties based on silk motifs. Historically, specific amino acid patterns have been linked to functional properties, and these patterns can be genetically or chemically modified to create recombinant silk polymers that control chemical reactivity, polymer size, and bulk material characteristics while preserving essential secondary structural features [91].

### 6.2. Wound Healing Mechanism of Silk Fibroin

**Figure 4:**
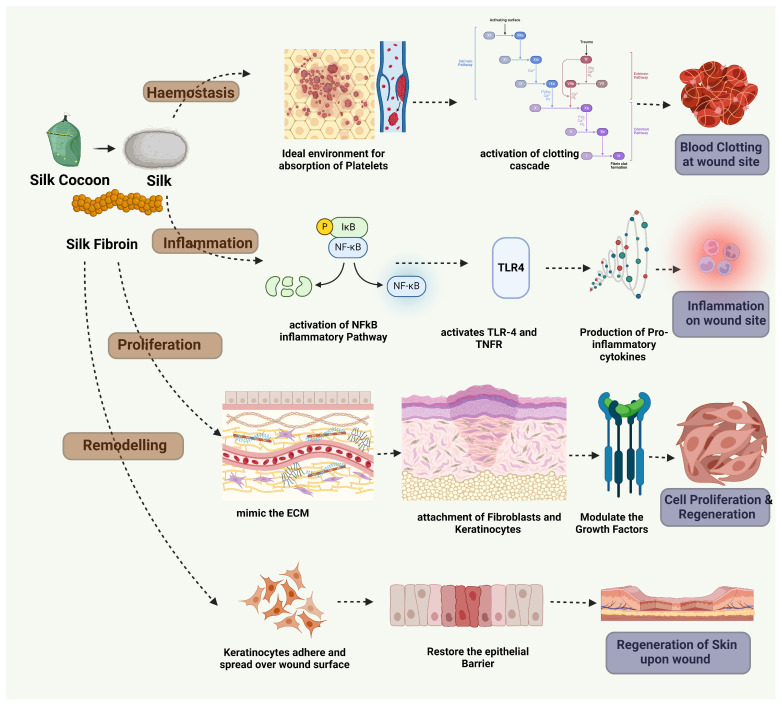
Silk fibroin aids wound healing by modulating inflammation, supporting angiogenesis, and enhancing ECM remodeling. It reduces inflammatory cytokines, promotes cell growth and migration, and improves ECM organization throughout the healing phases.

Silk fibroin (SF) plays a pivotal role in various stages of the wound healing process, demonstrating its effectiveness across multiple mechanisms as clearly described in Figure 4. One of the key contributions of SF is in promoting hemostasis, the initial and crucial step in healing. The structure of SF provides an ideal environment for the rapid adsorption of platelets, leading to the activation of the coagulation cascade and subsequent clot formation. This is facilitated by the porous nature of SF matrices, which efficiently trap blood cells and platelets, thereby accelerating the process of hemostasis. By minimizing blood loss and creating a stable environment at the wound site, SF sets the stage for the healing process to proceed smoothly [92,93,94]. As healing progresses, SF continues to support critical cellular behaviors such as attachment, migration, and proliferation. Its biochemical and physical properties mimic the extracellular matrix, promoting integrin-mediated cell adhesion. This encourages the attachment of various cell types, including keratinocytes and fibroblasts, to the wound bed, while also guiding cell migration—a process essential for wound closure. Additionally, SF modulates the release of growth factors and cytokines, enhancing cellular proliferation and ensuring effective tissue regeneration. The impact of SF extends further as it supports neovascularization and angiogenesis, processes vital for supplying nutrients and oxygen to the healing tissue. SF matrices, with their porous structure and biodegradability, provide a conducive environment for endothelial cell infiltration and new blood vessel formation [95,96,97]. By facilitating the release of angiogenic factors like VEGF in a controlled manner, SF enhances the formation of a capillary network, ensuring the regenerating tissue receives an adequate blood supply. This vascular support is crucial for effective healing, preventing tissue necrosis and promoting overall tissue health [98]. SF plays an instrumental role in re-epithelialization, the phase of wound healing that restores the epidermal barrier. The biocompatibility and structural mimicry of natural ECM by SF enable keratinocytes to adhere, spread, and form a new epithelial layer over the wound. By maintaining an optimal moisture balance and protecting the wound from external contaminants, SF creates an ideal microenvironment for re-epithelialization, speeding up wound closure and improving the quality of the regenerated skin [99].

The mechanisms by which silk fibroin enhances wound healing involve several intricate cellular signaling pathways. Silk fibroin accelerates wound healing primarily by modulating the NF-κB signaling pathway, a critical regulator of numerous cellular processes essential for tissue repair. NF-κB, or nuclear factor kappa light-chain-enhancer of activated B cells, is a transcription factor that controls the expression of genes involved in various cellular behaviors such as proliferation, adhesion, the clearance of reactive oxygen species (ROS), and inflammation. When silk fibroin interacts with cells, it stimulates the NF-κB signaling pathway by increasing the expression of receptors such as tumor necrosis factor receptor (TNFR) and Toll-like receptors (TLRs). These receptors, upon activation, initiate a cascade of intracellular events that lead to the activation of NF-κB. Once activated, NF-κB translocates into the cell nucleus, where it binds to specific DNA sequences to regulate the transcription of a broad array of genes. These genes encode proteins that play vital roles in the wound healing process [100]. For instance, NF-κB upregulates the production of cytokines and growth factors such as EGF and VEGF, which are essential for promoting cell proliferation and new blood vessel formation. Additionally, NF-κB enhances the expression of adhesion molecules like fibronectin, which are crucial for cell attachment and migration, facilitating the re-epithelialization of the wound. Moreover, NF-κB’s role in controlling the inflammatory response is particularly significant in wound healing. By regulating the production of pro-inflammatory cytokines like interleukin-1 beta (IL-1β) and anti-inflammatory cytokines like interleukin-10 (IL-10), NF-κB helps balance the inflammation process, ensuring that it is sufficient to combat infection without causing excessive tissue damage. This balance is vital for clearing the wound of pathogens and debris while allowing tissue regeneration to proceed efficiently. Furthermore, NF-κB influences the production of proteins such as vimentin, which is involved in cell shape and movement, and transforming growth factor-beta (TGF-β), which is key in tissue remodeling and scar formation [101]. By orchestrating these complex cellular responses, NF-κB, activated by silk fibroin, plays an integral role in advancing the wound healing process from the initial inflammatory phase through to the proliferation and remodeling stages. These processes are vital for effective wound healing, particularly in diverse types of wounds such as corneal epithelial wounds. In silk fibroin-induced cells, there is an upregulation of key mediators of NF-κB, including tumor necrosis factor receptor (TNFR) and Toll-like receptors (TLRs). By influencing the expression of these proteins, silk fibroin plays a critical role in the remodeling and proliferation phases of wound healing [102].

Moreover, silk fibroin has been shown to have protective effects in various models of injury. For example, in a rat model of burn injury, silk fibroin was found to inactivate the apoptotic pathway, thereby protecting cells from programmed cell death. This protective effect is likely mediated through the regulation of several complex cellular signaling pathways, including Wnt and Notch signaling, TGF-β signaling, mitogen-activated protein kinase (MAPK) signaling, and AKT/mTOR signaling [103]. Both MAPK and AKT/mTOR pathways are well-known for their roles in wound healing, where they contribute to cell proliferation, migration, and survival [104]. The ability of silk fibroin to modulate critical signaling pathways, particularly NF-κB, along with its biocompatibility, user-friendly nature, and minimal immune response, positions it as a highly promising material for wound healing applications.

Silk fibroin can be utilized in various forms, including solutions, films, electrospun nanofiber mats, hydrogels, hydrocolloid dressings, and sponges, to enhance wound healing. Among these various forms, electrospun nanofiber mats stand out as the most promising option due to their unique properties and superior performance in enhancing wound healing. This brings us to a closer examination of the electrospinning technique and its advantages in the development of silk fibroin-based wound dressings. However, silk fibroin can be formed using several other methods, each with its own set of advantages and limitations. To provide a comprehensive overview, the following Table 3 outlines the benefits and drawbacks of various methods for forming silk fibroin. Additionally, 3D printing is emerging as an innovative technique that holds great potential for creating wound-healing materials. 3D Printing provides superior control over scaffold architecture, enabling precise customization in terms of pore size, shape, and spatial distribution, which can enhance the material’s interaction with cells and improve wound healing. Electrospinning and freeze drying, while established methods, lack the same level of control over scaffold structure. Additionally, 3D printing eliminates the need for potentially harmful solvents, making it a more biocompatible option for some applications. However, 3D printing can struggle with mechanical strength, similar to hydrogel methods, and its use in processing silk fibroin still requires further optimization to match the efficiency of established methods like electrospinning [105].

## 7. Electrospinning—Fabrication Method of Silk Fibroin

### 7.1. Electrospinning

Electrospinning has emerged as a prominent technique for the fabrication of nanofibers, especially in the field of biomedical applications such as wound healing. Among the various methods available for nanofiber preparation such as drawing, phase separation, template synthesis, extraction, and self-assembly [106]. Electrospinning is considered the most feasible and straightforward. The primary advantages of electrospinning over other methods include its ability to produce nanofibers at a large scale, the use of a wide range of polymers, and the elimination of the need for post-synthetic processing, which are often required in other techniques [107]. This method has seen significant advancements in recent years, particularly for applications in diabetic wounds (DWs) [108]. Electrospinning operates on a simple yet highly effective principle. A polymer solution or melt is placed within a syringe equipped with a needle, which serves as the spinneret. An electric potential is then applied to this polymer solution, resulting in the formation of fibers with diameters ranging from 50 to 1000 nm or even greater. The process begins with the polymer solution being extruded through the needle, forming a droplet at the tip. Under the influence of the applied electric field, this droplet elongates, creating a cone shape known as the Taylor cone. As the electric potential increases, it reaches a critical point where the electrostatic forces overcome the surface tension of the polymer solution, leading to the ejection of a fine jet of the solution. This jet undergoes rapid stretching and thinning due to solvent evaporation and electrostatic forces, ultimately forming nanofibers that are collected on a stationary or rotating metal collector [109,110]. The electrospinning setup is relatively simple and typically consists of three main components: a high-voltage power supply, a syringe with a needle, and a conductive collector. The high-voltage supply provides the necessary driving force to generate an electric field between the spinneret and the collector. The polymer solution, housed within the syringe, is fed at a controlled rate toward the needle tip, where it is subjected to the electric field. As the polymeric jet is ejected from the Taylor cone, it travels towards the collector, where the solvent evaporates, leaving behind solid nanofibers [111]. Several key parameters influence the electrospinning process and the resulting nanofiber morphology. They are 1. Applied Voltage: The strength of the electric field directly affects the formation of the Taylor cone and the ejection of the polymer jet. Higher voltages can increase the rate of fiber production but may also lead to defects such as beading if not carefully controlled [112]. 2. Solution Flow Rate: The rate at which the polymer solution is fed through the needle influences the thickness and uniformity of the fibers. A higher flow rate can result in thicker fibers, while a lower flow rate produces finer fibers. 3. Needle Diameter: The internal diameter of the needle determines the initial diameter of the polymer jet and, consequently, the final Fiber diameter. A smaller needle diameter generally leads to finer Fibers. 4. Tip-to-Collector Distance: The distance between the needle tip and the collector affects the time available for the jet to stretch and the solvent to evaporate. A longer distance allows for more thinning of the fibers, while a shorter distance may lead to incomplete solvent evaporation and thicker fibers. 5. Ambient Conditions: Temperature, humidity, air velocity, and pressure within the electrospinning chamber play significant roles in the process [113,114]. For example, higher humidity can cause the fibers to retain moisture, leading to defects, while temperature fluctuations can affect the viscosity of the polymer solution and the rate of solvent evaporation.

Electrospinning has a rich history, dating back to its first patent in 1934 by Anton Formhals, who originally intended it for textile production. The technique was initially used to produce threads, which were collected and converted into fabric using a ground collector. Over time, electrospinning has evolved into a powerful method for producing nanoscale fibers, particularly for biomedical applications. The technique gained prominence in the late 20th and early 21st centuries as researchers recognized its potential for creating nanofibers that closely mimic the extracellular matrix, an essential component in tissue engineering and wound healing [115,116], Table 4 compared and contrasted the role of ECM and electrospun mats in the process of wound Healing. In the context of wound healing, especially for diabetic wounds, electrospun nanofibers offer several advantages. These fibers have a high surface-to-volume ratio, which enhances cell attachment and proliferation, crucial for tissue regeneration. The porosity of the nanofibrous scaffolds allows for adequate nutrient and oxygen exchange while providing a barrier against bacterial invasion. Furthermore, the chemical versatility of electrospun fibers enables the incorporation of bioactive molecules, such as growth factors or antimicrobial agents, to promote faster and more effective wound healing. The recent advancements in electrospinning have enabled the production of nanofibers with diverse structural and morphological organizations, tailored to meet specific biomedical applications. For example, researchers have developed core-shell nanofibers for controlled drug release, aligned nanofibers to guide cell growth, and multilayered scaffolds that mimic the complex structure of natural tissues. These innovations highlight the potential of electrospinning as a standard method for fabricating nanofibers in regenerative medicine. Electrospinning is a highly adaptable and efficient technique for producing nanofibers with a wide range of applications, particularly in wound healing. Its ability to control fibers morphology and tailor the properties of nanofibers to specific needs has made it a cornerstone in the development of advanced biomaterials [117,118]. As research in this area continues to progress, electrospinning is likely to play an increasingly important role in creating innovative solutions for medical challenges, such as the treatment of diabetic wounds.

### 7.2. Types of Electrospinning

As a “top-down” fiber-production technique, electrospinning offers several advantages, including simplicity in operation, low production costs, and minimal energy consumption. This method effectively combines polymer materials with inorganic nanoparticles to produce fibers with diameters in the nano- to micrometer range relatively quickly. The earliest and most commonly used electrospinning system is the single-spinneret system. Over time, advancements in technology have led to the development of more complex systems, such as double- and multineedle configurations. Researchers have also tailored nanofibers with specific shapes and structures by varying parameters such as polymer type, molecular weight, solution conductivity, voltage, flow rate, temperature, and humidity. These adjustments can produce a range of fiber structures, including porous, pleated, hollow, beaded, and reticulated forms, making electrospinning a prominent research area in various fields [119]. Uniaxial electrospinning, also known as single-fluid or traditional electrospinning, is one of the most widely utilized techniques for fabricating nanofibers. This process involves using a single polymer solution or melt, which is extruded through a spinneret under the influence of a high electric field [120]. Due to the limitations of single-fluid electrospinning, multifluid electrospinning techniques have been developed. These methods involve two or more working fluids, allowing for the creation of more complex nanofiber structures. Multifluid electrospinning includes coaxial electrospinning, side-by-side electrospinning, triaxial electrospinning [121,122,123,124].

### 7.3. Mechanism of Wound Healing by Electrospun Fibers in the Various Phases of Wound Healing

Electrospun fibers are proving to be transformative in wound healing due to their versatile and beneficial properties across various phases of the healing process. In the initial Hemostasis phase, which is crucial for stopping bleeding and forming a stable clot, electrospun fibers provide essential mechanical support [125]. Their highly porous structure aids in trapping blood cells and platelets, thereby facilitating effective clot formation. This early intervention helps stabilize the wound environment, minimizing excessive bleeding and creating a foundation for the subsequent phases of healing. During the inflammation phase, which focuses on cleaning the wound and initiating repair processes, electrospun fibers play a significant role in modulating the inflammatory response [126]. Many of these fibers are designed to incorporate anti-inflammatory agents or possess intrinsic properties that help mitigate excessive inflammation, which is critical as prolonged inflammation can delay healing. The fibrous network also supports the migration of inflammatory cells, such as macrophages and neutrophils, to the wound site. This migration is essential for clearing out cellular debris and pathogens. Additionally, the fibers can be engineered to release growth factors gradually, which aids in controlling inflammation and preparing the wound bed for the next phase of healing. As the wound transitions into the proliferation phase, electrospun fibers provide a scaffold that is pivotal for cellular activities such as fibroblast and keratinocyte proliferation and migration. The fibers micro and nanostructures closely mimic the extracellular matrix (ECM), offering a supportive environment that guides the orientation and growth of these cells. This mimicry is crucial as it helps in forming new tissue layers [126]. Furthermore, electrospun fibers enhance angiogenesis—the formation of new blood vessels—by providing a conducive environment for vascular growth. This process is vital for supplying the growing tissue with necessary nutrients and oxygen. In addition, the fibers can be tailored to support collagen deposition, which is key for reinforcing tissue strength and integrity. In the final remodeling phase, which involves the maturation and reorganization of the newly formed tissue, electrospun fibers contribute significantly to tissue development [127]. By replicating the ECM, these fibers help in the proper alignment and maturation of collagen fibers, which is crucial for enhancing tissue strength and elasticity. Biodegradable electrospun fibers are particularly advantageous as they degrade in a controlled manner, aligning with the natural timeline of ECM replacement. This controlled degradation helps in minimizing scar formation and ensuring the stability of the newly formed tissue [128].

### 7.4. Electrospun Silk Based Scaffolds from Bench to Bed Side

The evolution of wound care and tissue engineering has brought forth numerous innovations, among which electrospun silk-based scaffolds stand as a significant milestone. These scaffolds have garnered widespread attention due to their unique combination of biocompatibility, biodegradability, and mechanical strength, which closely mimics the extracellular matrix (ECM) of human tissues. The electrospinning process, a versatile technique capable of producing nanofibers with high surface area-to-volume ratios, plays a crucial role in enhancing the functional properties of silk fibroin. This has led to the development of advanced wound dressings and tissue scaffolds that support cellular adhesion, proliferation, and differentiation. Despite these challenges, the potential benefits of electrospun silk-based scaffolds are immense. Their ability to deliver bioactive agents, such as growth factors and antimicrobial peptides, in a controlled and sustained manner is particularly advantageous for treating chronic wounds, such as those associated with diabetes.

**Figure 5:**
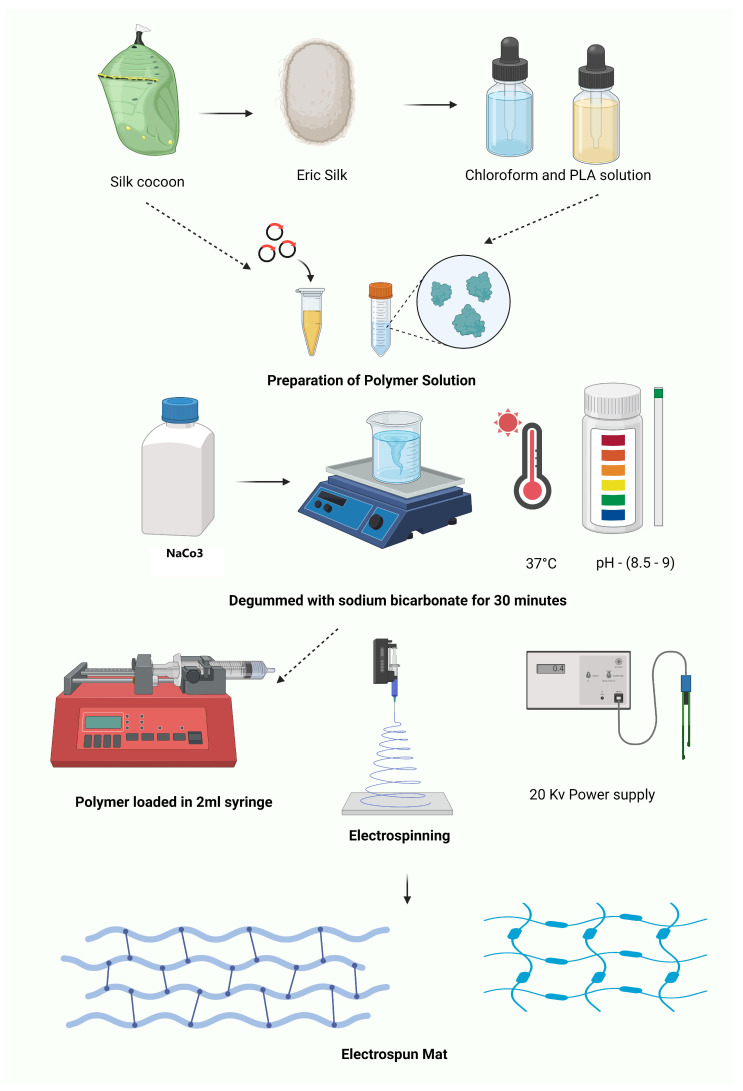
The electrospun mat formulation by Kuberasampathkumar Shanmugam and Subramanian Sundaramoorthy involves dissolving Eri silk fibroin and PLA in suitable solvents, blending the solutions, and electrospinning the blend into nanofibers. The resulting mat is dried and characterized for its properties using techniques such as SEM and tensile testing to ensure its suitability for wound dressing applications [135].

The synthesis of silk-based dressings and the application of electrospinning techniques converge to form a powerful strategy in wound management. By harnessing the natural biocompatibility and structural integrity of silk fibroin and combining it with the versatility of electrospinning, researchers have developed electrospun silk-based scaffolds that offer a unique blend of properties ideal for wound healing. These advanced dressings not only capitalize on the inherent advantages of silk fibers but also leverage the fine-tuned control over fiber morphology, porosity, and surface functionality provided by electrospinning. The resulting electrospun silk-based dressings represent a sophisticated integration of material science and bioengineering, designed to meet the complex demands of wound repair and tissue regeneration. In the study conducted by K. Karthikeyan et al. [129] novel biofibers were fabricated by coating silk fibroin with chitosan impregnated with silver nanoparticles, resulting in scaffolds that exhibited potent antibacterial properties and enhanced wound healing potential. Similarly, Salvador Aznar-Cervantes et al. [130] developed electrospun silk fibroin scaffolds coated with graphene oxide and reduced graphene, which demonstrated improved electrical conductivity, mechanical strength, and biocompatibility, crucial for promoting cell adhesion and proliferation in wound healing. Sónia P. Miguel et al. [131] produced and characterized electrospun silk fibroin-based asymmetric membranes, which featured a porous top layer for moisture management and a denser bottom layer for structural support, thus creating an ideal wound environment that promotes healing while preventing infection. Additionally, M. Rama and U. Vijayalakshmi [132] investigated the influence of silk fibroin in nanofibrous scaffolds designed for osteoregenerative applications, revealing enhanced osteoconductive properties that could be particularly beneficial in cases where bone exposure is involved in deep wound healing. Win chun Juo et.al [133,134] focused on the fabrication and characterization of electrospun silk fibroin/TiO_2_ nanofibrous mats, where the inclusion of TiO_2_ was shown to improve mechanical properties, enhance cell proliferation, and provide antibacterial activity, making these mats promising candidates for wound dressings. Kuberasampathkumar Shanmugam and Subramanian Sundaramoorthy [135] explored the development of electrospun mats from Eri silk fibroin and PLA blends, highlighting their balanced mechanical strength, biodegradability, and biocompatibility, which support cell proliferation and effective wound healing as shown in Figure 5. These studies collectively underscore the versatility and effectiveness of electrospun silk fibroin-based scaffolds in various wound healing applications, from antibacterial treatments to supporting tissue regeneration.

In recent years, the integration of electrospun silk-based scaffolds into clinical practice is a rapidly advancing field. Ongoing research aims to optimize their properties, explore their multifunctional capabilities, and establish standardized protocols for their use in various medical applications. As these scaffolds continue to move through the pipeline from experimental models to clinical trials, they represent a beacon of hope for patients suffering from non-healing wounds and other tissue defects. The application of biomaterials as wound dressings for diabetic wounds has been extensively studied in Clinical research settings. However, only a limited number of these have progressed to clinical testing, and even fewer have received approval and have become commercially available for diabetic wound management in the past five years. Several barriers hinder the clinical translation of these wound dressings from research to market. One significant challenge is the necessity for additional validation by regulatory bodies such as the U.S. Food and Drug Administration (FDA) or the European Union Medical Device Regulation when chemical modifications are made to biomaterials to enhance their inherent properties. This requirement introduces an extra step in the process of material safety and quality assessment, which must be completed before the already time-intensive technology validation can begin. Moreover, the standard methods used to characterize traditional wound dressings may not be sufficient to demonstrate the advanced capabilities of innovative wound dressings in enhancing wound healing. More sophisticated models of diabetic wound conditions and more comprehensive techniques are often needed to simultaneously assess tissue regeneration rates, drug release efficiency, and stimuli-responsive mechanisms. In addition, the complexity of these advanced wound dressings, often featuring multicomponent and multifunctional cores, complicates the processes of device classification and validation. It can be challenging to determine the primary mode of action of a wound dressing, given that many exhibits combined mechanisms.

## 8. Conclusions and Future Perspectives

The clinical translation of electrospun silk fibroin-based formulations holds great promise but faces significant challenges, including the scalability of production, regulatory hurdles, and the need for thorough safety and efficacy evaluations. Overcoming these obstacles will require collaborative efforts and rigorous testing to ensure these innovative biomaterials can be effectively and safely integrated into medical practice. Looking ahead, the future of electrospun silk fibroin-based formulations appears bright. Ongoing advancements in biofabrication techniques, along with a deeper understanding of silk fibroin’s interactions within biological systems, are expected to drive the development of next-generation biomaterials. These innovations have the potential to revolutionize medical treatments, offering personalized and highly effective therapeutic solutions that could significantly impact the field of regenerative medicine.

## Figures and Tables

**Table 1:** Role of various growth factors in the wound healing process.

S.No.	Growth Factors	Sources of Growth Factors	Function
1.	EGF	Platelets and macrophages	Re-epithelialization; granulation tissue formation
2.	PDGF	Platelets; keratinocytes; macrophages; endothelial cells; fibroblasts	Inflammation; granulation tissue formation/angiogenesis; re-epithelialization; matrix formation and remodeling
3.	TGF-β	Platelets; keratinocytes; macrophages; lymphocytes; fibroblasts	Inflammation; granulation tissue formation; re-epithelialization; matrix formation and remodeling
4.	TNF-α	Neutrophils; macrophages	collagen expression; re-epithelialization
5.	VEGF	Platelets; neutrophils; macrophages; endothelial cells; smooth muscle cells; fibroblasts	granulation tissue formation/angiogenesis/neo-vascularization
6.	FGF	Macrophages, fibroblasts, endothelial cells	Angiogenesis, granulation tissue formation, re-epithelialization, collagen synthesis
7.	IGF	Platelets, macrophages, fibroblasts	Re-epithelialization, matrix formation and remodeling, cell migration
8.	KGF	Fibroblasts	Stimulates keratinocyte proliferation and migration, re-epithelialization
9.	HGF	Mesenchymal cells, fibroblasts	Promotes epithelial and endothelial cell proliferation, re-epithelialization, angiogenesis

The table outlines the key growth factors involved in the wound healing process, highlighting their primary sources and functions. Epidermal growth factor (EGF), platelet-derived growth factor (PDGF), transforming growth factor-beta (TGF-β), tumor necrosis factor alpha (TNF-α), vascular endothelial growth factor (VEGF), fibroblast growth factor (FGF), insulin-like growth factor (IGF), keratinocyte growth factor (KGF), and hepatocyte growth factor (HGF).

**Table 2:** Synthetic polymer formulations and their effects on wound healing in experimental animals.

S.No.	Synthetic Polymers	Formulation	Experimental Animals	Highlights
1.	Polyethylene Glycol (PEG)	Chitosan crosslinked PEG Hydrogels loaded with silver nanoparticles	Rabbits	High porosity and improved antioxidant property and antimicrobial property. Boosted wound healing in experimental animals [44]
		Quaternized chitosan Combined PEG-CHO hydrogels	Rats	Good mechanical properties and Biocompatibilty, and improved angiogenesis and accelerated the process of wound healing in experimental animals [45]
		PEG-PDLLA Hydrogels	Mice	Enhanced angiogenesis and decreases the ROS, IL-6 and TNF. Promotes the healing of wounds [46]
2.	Polycaprolactone (PCL)	Composite PCL/electrospun nanocoated scaffolds with ACP and Si	Diabetic mice	Better mechanical stability, enhanced wound healing of full thickness wound by stimulating angiogenesis, collagen deposition and re-epithelialization [47]
		Composite nanofibrous scaffolds made of chitosan, gelatin, PCL, PVP	Diabetic rats	Cytocompatibility with respect to L929 cells. Accelerated the wound healing process by improving the collagen synthesis, lowering the inflammatory cytokines TNF, IL-6, IL-1 and NFkB [48]
3.	Polylactic acid (PLA)	Multilayered nanofibrous PLA patches	Diabetic rats	Better wound healing activity by adequate fibroblasts proliferation, angiogenesis and resulting in a healing of full thickness wound [49]
		Electrospun fibers made of PLA and PVP	Diabetic mice	Possesses anti-inflammatory and antibacterial activity prompting wound healing process [50]
4.	Poly(lactic-co-glycolic acid) (PLGA)	Composite PLGA/CNC nanofiber	Diabetic mice	Better cytocompatibility by fibroblast adhesion and accelerated full thickness wound healing decreasing the expression of IL-6 and IL-1B [51]
		PLGA incorporated nanoparticles	Diabetic mice	Very beneficial against MRSA diabetic wounds possess better wound healing efficiency [52]
5.	Polyvinyl alcohol (PVA)	PVA incorporated nanofibers	Diabetic mice	Accelerated closure of full-thickness Wounds in diabetic mice, with Enhanced revascularization and re-epithelialization, increased collagen deposition and remodeling, and improved hair follicle regeneration [53]
		PVA/GO hydrogels	Fibroblast cell lines	Good cytocompatibility and antibacterial activity and promote better wound healing activity [54]
6.	Polyvinyl pyrrolidone (PVP)	Composite nanofibrous scaffolds made of chitosan, gelatin and PVP	Diabetic rats	High tensile strength, accelerated wound healing by improving the collagen remodelling by decreasing the levels of TNF, IL-6 and NFKB [55]
		PVP-grafted microspheres	Diabetic mice	Antibacterial and accelerated wound healing activity in experimental animal [55]

This table summarizes the various synthetic polymer formulations and their experimental applications in wound healing studies. The abbreviations used represent key compounds and biological markers relevant to the study, including polyethylene glycol (PEG), aldehyde group (CHO), poly(D,L-lactic acid) (PDLLA), polycaprolactone (PCL), amorphous calcium phosphate (ACP), silicon (Si), polyvinyl pyrrolidone (PVP), polylactic acid (PLA), Poly(lactic-co-glycolic acid) (PLGA), cellulose nanocrystals (CNC), methicillin-resistant *Staphylococcus aureus* (MRSA), polyvinyl alcohol (PVA), graphene oxide (GO), tumor necrosis factor, interleukin 6 (IL-6), interleukin 1 (IL-1), and nuclear factor kappa B (NF-κB).

**Table 3:** Comparison of various methods for forming silk fibroin and their application in wound healing [105].

S.No.	Methods	Advantages	Disadvantages
1.	Electrospinning	Produces nanofibers with high surface area Mimics extracellular matrix (ECM) Easy functionalization	Requires volatile solvents Limited fiber control and alignment
2.	Freeze Drying	Creates porous structures Good for tissue scaffolds Retains bioactivity	Fragile structures Time-consuming process
3.	Solvent Casting	Simple and inexpensive Suitable for film production Good control over film thickness	Limited pore structure Residual solvent toxicity issues
4.	Hydrogel Formation	Excellent moisture retention Mimics ECM Ideal for drug delivery systems	Poor mechanical strength Limited scalability
5.	Lyophilization	Suitable for highly porous scaffolds No need for organic solvents	Slow process High cost Can cause structural collapse
6.	3D Printing	Precise control over geometry and pore size Customizable designs No need for toxic solvents	Limited mechanical strength in some cases Requires optimization for silk fibroin processing

This table compares methods for forming silk fibroin in wound healing. Each method has strengths, like the high surface area of electrospun nanofibers and the precision of 3D printing, but also limitations such as solvent use and scalability.

**Table 4:** Comparison of electrospun nanofiber scaffolds and extracellular matrix.

Comparison	Extracellular Matrix (ECM)	Electrospun Scaffolds
Components	Comprised of a dynamic 3D network of polysaccharides and natural polymers (collagen, elastin, fibrinogen, etc.)	Primarily made from synthetic and natural polymers
Structure	Consists mainly of fibers with diameters between 50 and 500 nm	Nanofibers typically range from 10 µm to 500 nm in diameter, produced via electrospinning
Role in Wound Healing	Facilitates pattern formation, morphogenesis, and phenotype maintenance during development Mediates clot formation, wound healing, inflammation, granulation tissue formation, and tissue remodeling Supports cell adhesion, migration, growth, differentiation, and apoptosis	Offers suitable porosity for cell migration and penetration Provides ample surface area and chemistry for cell adhesion, growth, migration, and differentiation Degrades at a rate that aligns with tissue regeneration, supporting proper tissue integration

The table provides a comparative overview of electrospun nanofiber scaffolds and the extracellular matrix (ECM) with respect to their components, structure, and roles in wound healing.

## Abbreviations

DM	Diabetes Mellitus
VEGF	Vascular Endothelial Growth Factor
TGF	Transforming Growth Factor
IL-4	Interleukin-4
IL-13	Interleukin-13
TNF	Tumor Necrosis Factor
FGF	Fibroblast Growth Factor
PLA	Polylactic Acid
PCL	Polycaprolactone
PLGA	Poly(lactic-co-glycolic acid)
PVA	Polyvinyl Alcohol
ECM	Extracellular Matrix
SF	Silk Fibroin
NFkB	Nuclear Factor-kappa B
ROS	Reactive Oxygen Species
TLR	Toll-Like Receptor
TNFR	Tumor Necrosis Factor Receptor
MAPK	Mitogen-Activated Protein Kinase
AKT	Protein Kinase B
IL-10	Interleukin-10
FGF	Fibroblast Growth Factor
IGF	Insulin Like Growth Factor
KGF	Keratinocyte Growth Factor
HGF	Hepatocyte Growth Factor
